# The ARUTIS Study (Anglia Ruskin University Trial of the Intuitive System): a single-centre, double-masked randomised controlled crossover trial of precision tinted lenses for visual stress: study protocol for a randomised controlled trial

**DOI:** 10.1186/s13063-025-09305-8

**Published:** 2025-12-16

**Authors:** Zahra N. Ramsahye, Peter M. Allen, Nikita J. Thomas, Arnold J. Wilkins, Bruce J. W. Evans, James M. Gilchrist

**Affiliations:** 1 Vision and Hearing Sciences Research Centre, Anglia Ruskin University, Cambridge, CB1 1PT United Kingdom; 2 Department of Psychology, University of Essex, Colchester, CO4 3SQ United Kingdom; 3 Optometry and Visual Science, City St George’s University of London, London, EC1V 0HB United Kingdom; 4 Unaffiliated, Independent researcher, Bradford, United Kingdom

**Keywords:** Visual stress, Intuitive system, Intuitive colorimeter, Colorimetry, Precision tinted lenses, Wilkins rate of reading test, Symptoms

## Abstract

**Background:**

Visual stress (VS), a condition affecting approximately 20% of children with reading difficulties that is alleviated with individually prescribed coloured filters, remains controversial. The purpose of the Anglia Ruskin University Trial of the Intuitive System (ARUTIS) is to investigate the effect of wearing precision tinted lenses (PTL) prescribed by the Intuitive System (IS) in children with VS using a double-masked crossover design.

**Methods:**

A total of 120 participants aged 9–18 years meeting diagnostic criteria for VS will be recruited from the Anglia Ruskin University Eye Clinic. A widely used instrument, the Intuitive Colorimeter, will be used to select, for each participant, the chromaticity of precision tinted lenses (PTL) that they claim optimally alleviates their symptoms and a similar control colour will also be determined. Participants will be randomised into group 1, receiving spectacles with intervention A (optimal tint) followed by B (sub-optimal tint), or group 2, receiving the two interventions in the order BA. The first intervention will be used for 1 month, followed by a 1-month washout period, the second intervention for 1 month, and then a head-to-head comparison in the clinic with participants invited to keep their preferred pair of spectacles. The primary outcome measurement is symptoms (measured by symptom diaries and symptom questionnaires); the secondary outcome measure is observed school performance (measured by academic behaviour surveys); and the tertiary outcome measure will be reading speed (measured by the WRRT).

**Discussion:**

Reviews on VS have reached contradictory conclusions, noting the need for stronger evidence. This trial will be the first in children to use modern diagnostic criteria and will strengthen the evidence base concerning the widespread but still controversial use of coloured filters for children with VS who struggle to read.

**Trial registration:**

Prospectively registered to clinicaltrials.gov on 17th October 2023 with a unique protocol ID of ETH2223-7100.

**Supplementary Information:**

The online version contains supplementary material available at 10.1186/s13063-025-09305-8.

## Background

Visual stress (VS) is a condition characterised by symptoms of eye strain, headache, and visual perceptual distortions when reading [1–7] that are reported to be alleviated by individually selected coloured filters [5, 8–15]. VS is controversial [8, 16, 17]. Much of the controversy may be explained by studies and reviews conflating VS with dyslexia. Evidence indicates that these are separate conditions that sometimes co-occur, with VS affecting approximately 20% of people with dyslexia [13]. There is literature that claims the treatment of VS with coloured filters is attributable to placebo effects [8, 16, 17] but also publications [1, 3, 5, 9, 13, 18, 19] asserting that coloured filters reduce symptoms of asthenopia and visual perceptual distortions that occur in people with VS. There is a consensus that further clinical trials are needed to evaluate the effect of colour on the symptoms of VS.

A neurological theory for VS was originally proposed in 1984 on the basis that the visual stimuli that evoke discomfort are generally those that also induce seizures in patients with photosensitive epilepsy [6, 20]. Striped patterns are particularly problematic, and text forms a striped pattern that can trigger symptoms [6]. A neural mechanism for VS has found support in studies showing that the visual stimuli that induce discomfort also induce a large haemodynamic response, both in absolute terms and relative to the response to comfortable stimuli [21]. Individuals who are particularly susceptible to discomfort exhibit an abnormally large haemodynamic response [22, 23].

This work led to the cortical hyperexcitability hypothesis for the aetiology of VS. If the cortex is hyperexcitable, it is likely that the hyperexcitability is not diffuse but patchy, as it is in photosensitive epilepsy, where small areas of the visual cortex that respond, for example, to certain orientations of lines can trigger seizures [7]. It has been argued that precision tinted lenses (PTLs) rearrange cortical activity in such a way as to avoid strong excitation in hyperexcitable regions [24]. The avoidance of strong excitation in hyperexcitable regions prevents the spread of excitation and, in doing so, prevents the inappropriate firing of visual neurons that gives rise to illusions and distortions [24].

In 1994, Wilkins et al. [5] carried out a randomised crossover trial (RCT) that aimed to detect whether PTLs relieve symptoms of VS when reading. For participants who completed this trial, symptomatic days were significantly less frequent with the experimental PTLs compared to the control PTLs.

However, this study has received criticism [20], as there was significant attrition, which could have led to an overestimation of the benefits from PTLs. Conversely, the selection criteria were broad, which may have underestimated the benefit of the PTLs by including participants who did not have VS. During the study, reading was assessed with The Neale Analysis of Reading Ability [20]. This test is used to detect reading age and comprehension levels in school-aged children and uses large text and widely spaced lines which means that the test is unlikely to detect a benefit from optometric interventions.

Subsequently, the Wilkins Rate of Reading Test (WRRT) [25] was developed with features that make it well-suited to investigating the effect of optometric interventions. This test has been widely used in research on VS and other optometric interventions [25, 26], but was not available at the time of the 1994 study [5, 25].

Two systematic reviews relating to VS were published in 2016 and reached different conclusions. A review by Evans and Allen [13] concluded that VS affects about 20% of people with reading difficulties and can be alleviated with coloured filters. The other review, by Griffiths et al. [20] concluded that the “use of coloured lenses or overlays to ameliorate reading difficulties cannot be endorsed” and argued that reported beneficial effects may be attributable to the placebo effect [6]. Both reviews agreed that this is a difficult area to research, and more trials are needed. Both note the limitations of the research and accept that there is not a high level of evidence. The different conclusions of the two reviews may be explained, at least in part, by the fact that the reviews asked different questions. Griffiths et al. ask whether coloured filters improve reading and therefore concentrate on reading skills. An earlier review concluded that coloured filters improve symptoms in VS but found that there is insufficient evidence to state whether they improve reading in those with reading difficulties [17]; therefore, the conclusion of Griffiths et al. was not unheralded. The Evans and Allen review concentrated on symptoms and, unlike Griffiths et al., focused on studies that used the scientifically designed Wilkins “Intuitive System” (IS) and, most importantly, studies of people with the target condition of VS [13]. The Griffiths et al. review included studies of populations with reading difficulties such as dyslexia, which would have been markedly underpowered because most people with dyslexia do not have visual stress [13].

The IS was developed by Wilkins at the MRC Applied Psychology Unit in the 1990s and has been widely used by eye care practitioners since then. It is the only system for treating VS that has been fully described in the scientific literature and that systematically samples human colour space [24]. The system comprises the Pattern Glare Test (PGT) [27], the WRRT [25], Intuitive Overlays (IOs) [9], Intuitive Colorimeter (IC), and PTLs [28]. It is important to note that clinical guidelines are that eye care professionals should carry out a comprehensive eye examination to rule out other causes of any symptoms before IOs or PTLs are prescribed [29].

Both systematic reviews published in 2016 noted that better diagnostic criteria are needed. In response, the following year, Evans et al. published a Delphi study to develop practical diagnostic guidelines for VS [2]. Two rounds of the Delphi process revealed that clinicians were using diagnostic criteria with reasonable consistency and led to diagnostic guidelines based on symptoms and signs, reproduced in Table 1.

**Table 1:** Diagnostic indicators of VS from the Delphi study by Evans et al. [2]

At least three of the following six typical symptoms:
1. Words move
2. Words merge
3. Patterns or shadows in text (e.g. “rivers”)
4. Text seems to stand out in 3-D above the page
5. Words or letters fade or darken
6. Discomfort with certain artificial lights and flicker
And
At least two of the following three signs from investigations:
1. Voluntary unprompted use of an overlay for 3 months or more
2. Overlay improves performance at the WRRT by ≥15%
3. PGT result >3 with mid-spatial frequency grating

Below, we present the study protocol for a double-masked randomised controlled crossover clinical trial (RCT). This RCT aims to investigate, in young people aged 9–18 years meeting contemporary diagnostic criteria for VS, whether PTLs prescribed using the IS are beneficial. The overall approach taken in the trial is to maximise the real-world relevance of the study by favouring a pragmatic approach, but not to an extreme extent that would invalidate the scientific rigour of the study.

### Objectives

- 1a. Assess recollected symptoms experienced during the day with symptom diaries maintained under the two different intervention conditions.
- 1b. Assess immediate symptoms experienced in response to viewing text, with symptom questionnaires presented under the two different intervention conditions.
- 2. Assess academic activity and behaviour by analysing surveys completed by parents/guardians and teachers under the two different intervention conditions.
- 3. Assess reading speed using the WRRT under the two intervention conditions.

## Design and methods

This will be a single-centre, double-masked randomised controlled crossover trial. There will be a 50:50 allocation ratio to evaluate the superiority of a hypothetically optimal intervention over a sub-optimal intervention.

### Study setting

All study testing will be carried out by a state-registered optometrist with extensive experience in this field at the University Eye Clinic, Anglia Ruskin University, CB1 2NN, UK. This clinic is appropriately equipped for the trial.

### Eligibility criteria

#### Inclusion criteria

To enter the study, participants must meet contemporary diagnostic criteria for VS, as summarised in Table 1. Further eligibility for the study is as follows:

1. Age 9–18 years.
2. Consent (parent/guardian and participant to attend optometric testing and participate in research)
3. Do not anticipate moving from the area in the next 3 months and happy to travel to the Anglia Ruskin University (ARU) eye clinic in Cambridge

In an audit by Evans et al. [30] of 323 participants consulting the first institutional clinic using the IC, the mean age was 14 years. This supports the observation that VS maximally affects secondary school children, who are “reading to learn” rather than “learning to read” [26]. This aligns with the theoretical explanation for VS outlined above: as children progress from large text with widely spaced lines at primary school to smaller more closely spaced text at secondary school, the risk of text possessing the spatial properties that can elicit symptoms increases [31]. It is likely that individuals with significant VS who are untreated will be less likely to continue to university than those without VS [9, 11]. Therefore, there will be a lower prevalence of untreated VS in the university population. It is also likely that the individuals with VS who progress to tertiary education will tend to be those with milder VS. Additionally, there is evidence that the sensitivity to striped stimuli that probably underlies VS on average decreases with age [27]. Therefore, children aged 9–18 years are likely to be a good age for a study of VS. Participants who meet the inclusion criteria will undergo optometric testing (see Supplementary Table 1, Additional file 1) by the lead researcher.

#### Exclusion criteria

Most practitioners who use the IC to prescribe PTLs are optometrists, and the most widely used professional guidance for this profession in the UK is that produced by the College of Optometrists [32]. This guidance recommends that before prescribing coloured filters, a full eye examination is undertaken to exclude other, more conventional, causes of symptoms. The proposed study will follow this advice, applying the clinical exclusion criteria in Supplementary Table 1, Additional file 1. The references in Supplementary Table 1, Additional file 1 are all widely cited clinical publications, and similar criteria are likely used in practice, although the criteria in Supplementary Table 1, Additional file 1 are likely to reflect “best practice”, which will not be followed by all clinicians. If potential participants exhibit any of the conventional optometric anomalies in Additional file 1 and, at least 1 month after that condition has been successfully treated, their symptoms remain and they still meet the diagnostic criteria for VS, then they can be started in the trial.

Further exclusion criteria are as follows:

1. History of wearing PTL.
2. Ocular pathology or systemic pathology that is likely to worsen in the timescale of the study or cause transient blur (e.g. diabetes) or photosensitive epilepsy

### Who will take informed consent?

Participants who are over the age of consent will complete their own signed consent. For participants under the age of consent (16 years old in the UK), their parent/guardian will complete consent, and assent will be obtained from the participant.

### Additional consent provisions for collection and use of participant data and biological specimens

There are no additional consent provisions for the collection and use of participant data. This study does not use biological specimens.

### Explanation for the choice of comparators

The proposed double-masked RCT compares an optimal and sub-optimal colour of PTL to investigate the effectiveness of the IS in reducing the symptoms of VS. The optimal PTL will be detected at the end of the trial, one which creates the least amount of VS symptoms, compared to the sub-optimal PTL which may not reduce VS symptoms to such a great extent.

### Intervention description

**Fig. 1:**
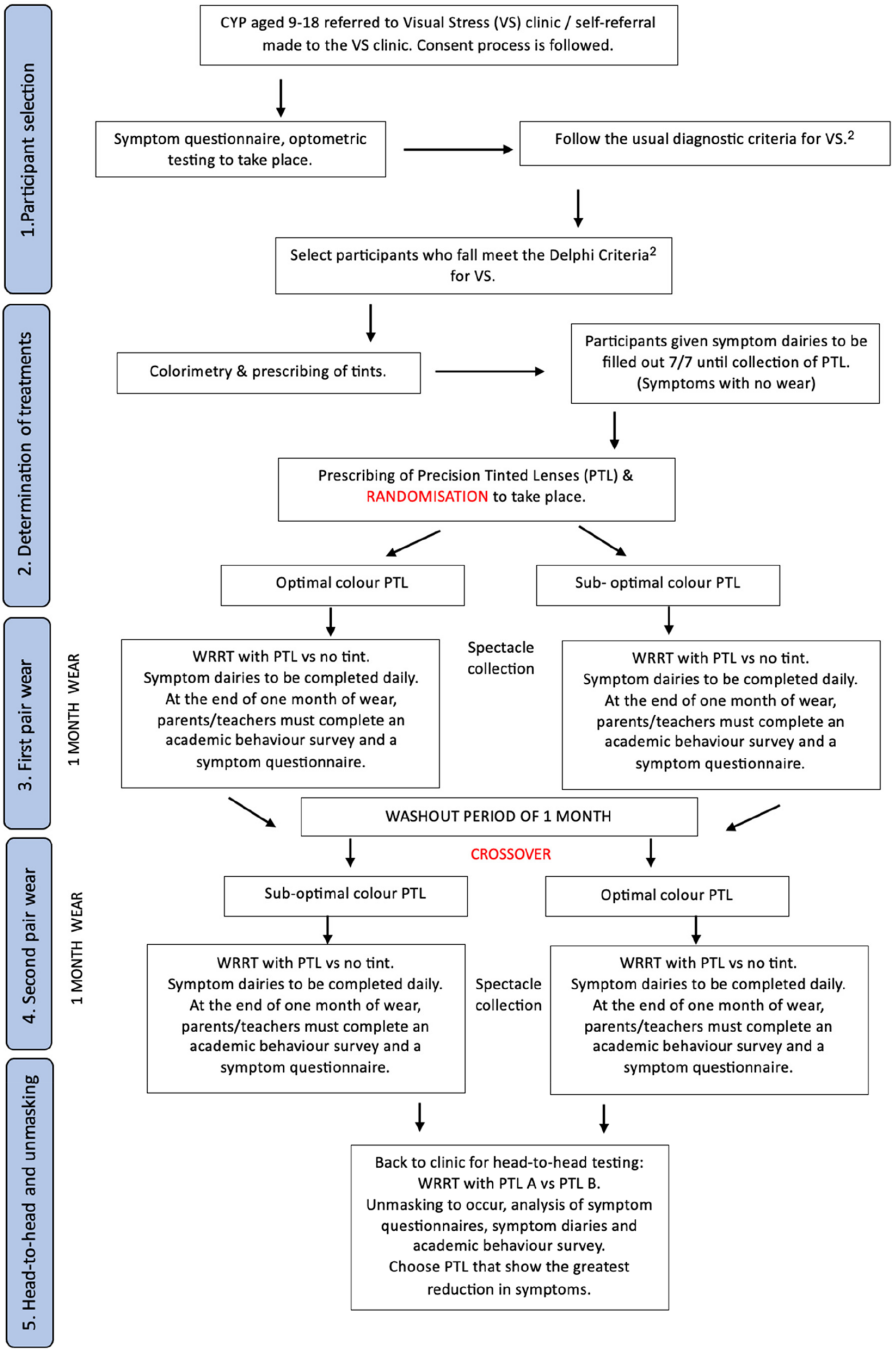
Participant flow diagram. CYP, children/young persons

The proposed double-masked RCT has five phases (Fig. 1). For a more detailed account of the procedure, please see Additional file 2.

1. Participant recruitment follows three stages (see Additional file 2).
2. Colorimetry, WRRT, and prescribing PTL interventions. Participants will be randomly allocated (with masking) to wear first either optimal (active) colour or sub-optimal (control) colour.
3. Short-term effects of the first pair of PTL:
  - Wilkins Rate of Reading Test (WRRT) at dispensing of first pair (first PTL vs no tint): The test will be administered (following test instruction manual) twice in each condition in the order ABBA with random allocation of PTL or no tint to A or B.
  - Symptoms with the first pair from diaries (see Additional file 5) throughout 1 month of wear and a questionnaire at the end of the month.
  - Academic behaviour survey completed by parents and teachers (see Additional file 6) in the last week of 1 month of wear.
4. After a washout period, each participant will crossover to the other pair, and the researcher will assess the short-term effects of the second pair of PTL, as described in (3) above, including WRRT with second PTL vs no tint in ABBA order.
5. After both pairs have been worn, participants will return for:
  - WRRT with first PTL vs second PTL in ABBA order with random allocation to A or B.
  - The researcher (maintaining masking) will compare symptom diaries from (3) and (4) and WRRT data to decide with the participant about the preferred pair to be dispensed to participants who wish to keep one pair.

### Criteria for discontinuing or modifying allocated interventions

If a participant considers the dispensed spectacle frames are unsuitable or uncomfortable, they can return to the researcher for frame adjustment or dispensing of more appropriate frames. In this scenario, the lens interventions will not be changed.

If a participant experiences aversion or discomfort with one of the interventions, they can stop wearing the PTLs but will be asked to record reasons in the symptom diary. Participants will have previously worn any new optical prescription for 1 month before entering the trial to ensure that there is no non-tolerance to the optical prescription (see Additional file 1).

The participant can remain in the trial, regardless of how much they wear their PTLs, as long as symptom diaries are completed. Written and verbal communication will be provided to participants explaining this when the spectacles are dispensed.

### Strategies to improve adherence to interventions

The lead researcher will take the following steps to minimise the likelihood of dropouts. Participants/carers will be reminded via SMS every 3 days to complete symptom diaries daily for 2 weeks. Following this, an SMS reminder will be sent out once a week. The lead researcher will also ensure that missed visits are rebooked at a convenient time for the participant. The lead researcher will encourage academic survey completion by sending out reminders via SMS and email (with consent) to parents and teachers at the end of 1 month of wear. The lead researcher aims to develop a positive rapport throughout the trial and explain the importance of adherence to the trial at the time of consent and subsequent interactions.

### Relevant concomitant care permitted or prohibited during the trial

Only one PTL intervention will be issued per month of wear and participants will be asked not to use any other coloured filters, coloured paper, or modifications to digital device display colour. Participants may carry on with their education and daily life, as PTL wear will not interfere with daily tasks, and PTL wear is instructed to be for reading tasks (but not necessarily only for reading tasks if participants wish to wear them for other tasks, such as television or computer games).

### Provisions for post-trial care

After the trial, participants will be offered the PTLs from the trial that are more beneficial and will be allowed to continue wearing these if they wish to. Participants will be encouraged to continue to receive regular optometric care as would be recommended had they not participated in the trial.

### Outcomes

#### Primary outcome measures

VS is defined as a condition characterised by symptoms [2], and a previous RCT [5] found symptoms to be the key outcome measure that demonstrated a benefit from individually prescribed PTL [5]. The clinical protocol for the detection, investigation, and management of VS starts with symptoms [26, 33]. Therefore, it is essential for pragmatic research on this topic to concentrate on symptoms. The first primary outcome measure will be symptom assessment using symptom diaries for each intervention. These symptom diaries were used by Wilkins et al. in the original RCT of PTL [5] (see Additional file 5). For the symptom diary, data will be divided into days and hours during which PTLs were worn and symptoms experienced.

The specific measurement variable will be symptom scores representing the number of symptomatic days with pair A vs pair B. The analysis will be carried out at the end of the 3-month trial, as participants will complete diaries throughout the trial.

**Fig. 2:**
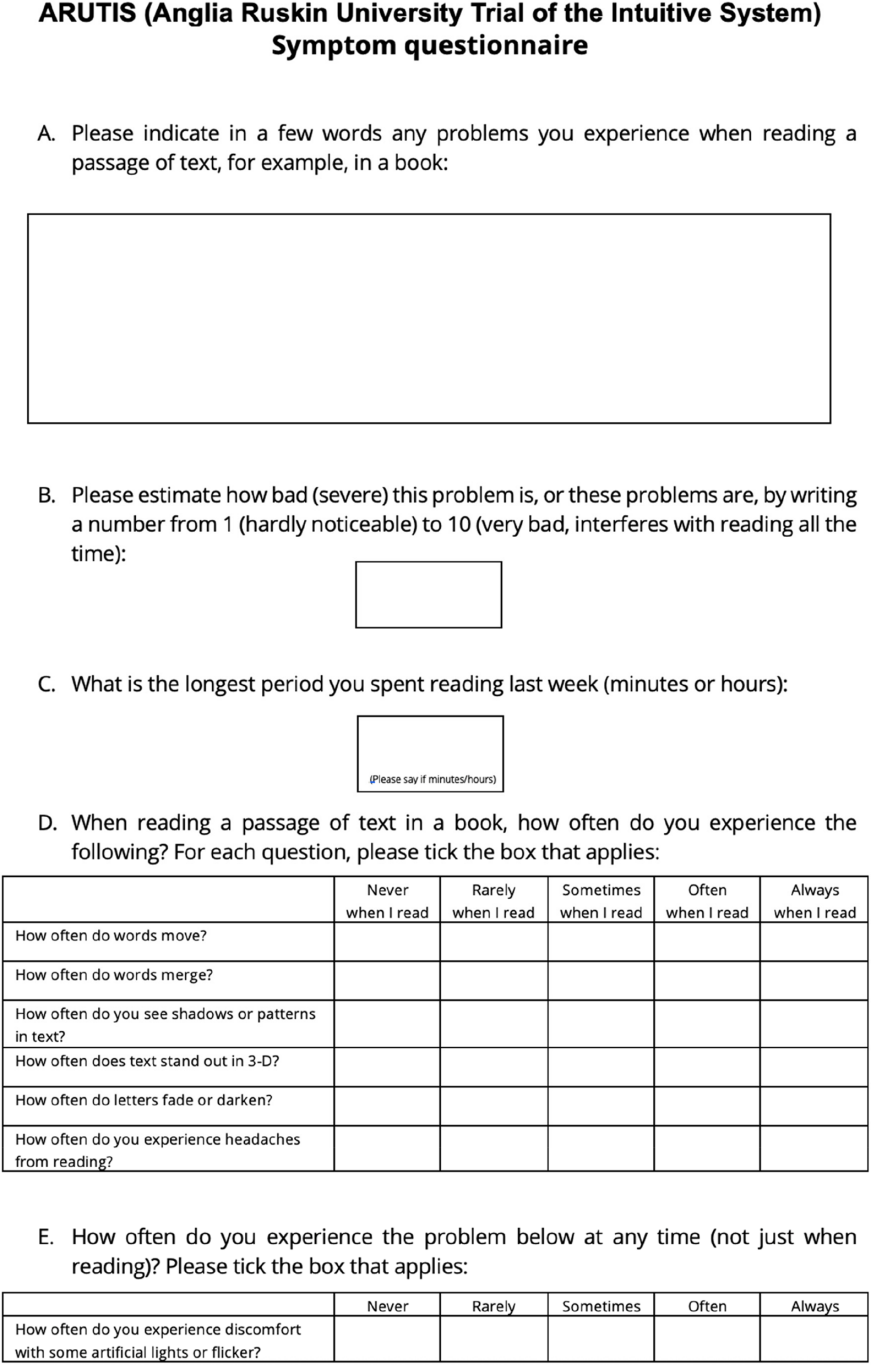
Symptom questionnaire

**Fig. 3:**
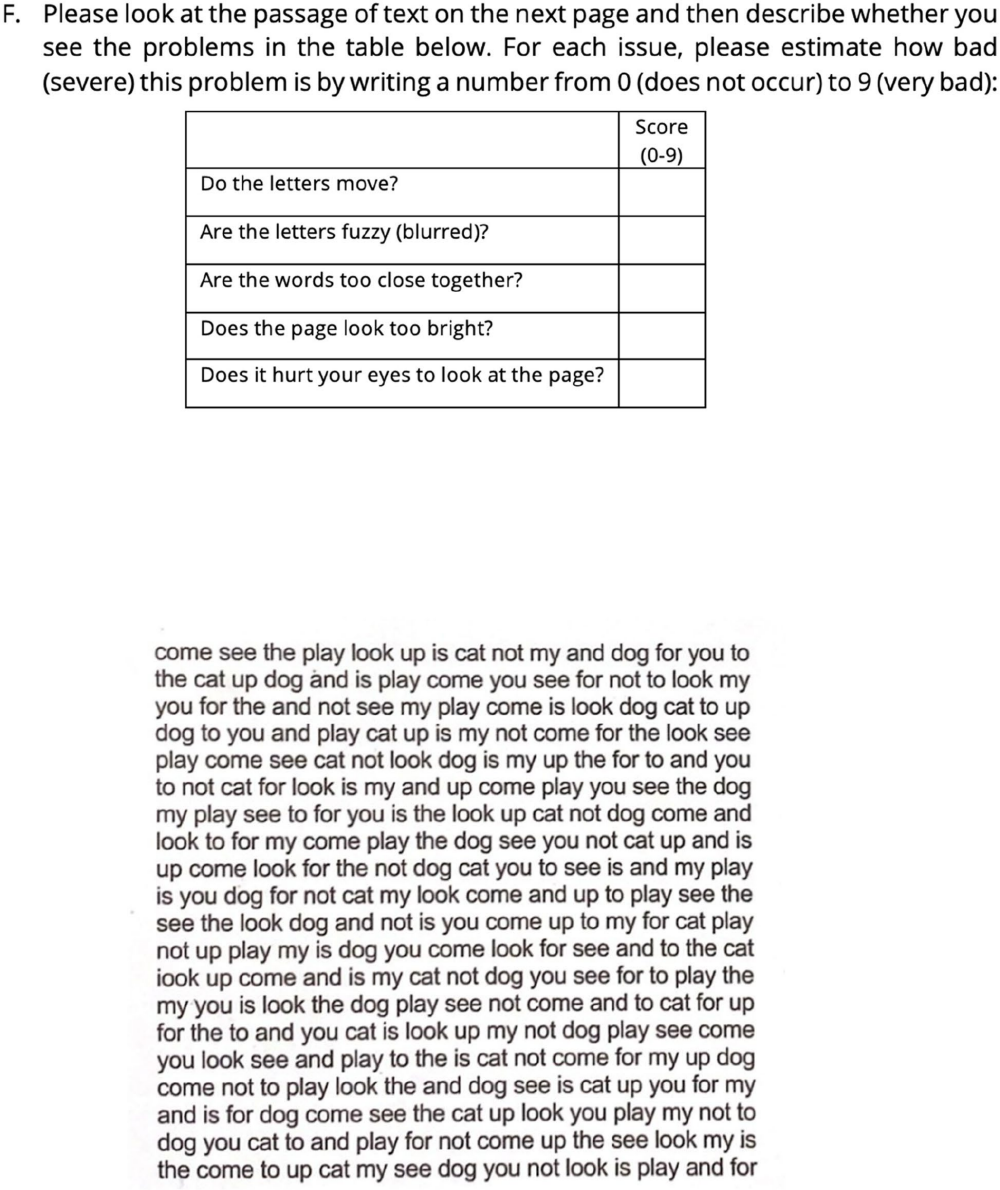
Symptom questionnaire

The second primary outcome measure will be a further assessment of symptoms, measured via symptom questionnaires administered at baseline (before interventions) and at the end of each wearing period (with each intervention). The symptom questionnaire (Figs. 2 and 3) presents participants with questions (A) to (F). At follow-up appointments, the questionnaire wording will be slightly different so that the initial questions assess changes in the originally reported prime symptom (see Additional file 10).

The two primary outcomes reflect fundamentally different methods of assessing symptoms. The daily diaries offer near real-time estimates of symptoms each day, providing a “live” assessment. In contrast, the questionnaires used at the end of each wearing period give a more long-term recollection of symptoms during that time and upon viewing a standardised passage of text through the relevant intervention.

The purpose of question (A) (Fig. 2) is to discover the presenting symptoms—what the participant considers to be their main problems when reading. In (B) (Fig. 2), the participant grades the severity of this symptom from 1 to 9. At follow-ups, participants will be asked to grade the same symptom again using the same scale, and this will be used to investigate changes with the interventions, with question (C) (Fig. 2) serving the same purpose (to investigate changes with the interventions) and to detect if there is a possible increase in the time spent reading with the interventions. Those who suffer from VS may avoid reading due to the presence of symptoms. Since this is the participant’s description of their main problems, it is clearly of utmost relevance to the participant and is therefore a suitable choice for a pragmatic trial.

The second part of the secondary outcome measures is addressed in questions (D), (E), and (F) (Fig. 3). Questions (D) and (E) (Fig. 3) ask participants questions according to the Delphi study. The questions about trait symptoms rely on the participant’s recollection of symptoms: trait symptoms are stimulus-specific, and reflect some of the Delphi [2] questions which also ask about the different aspects of the stimulus.

Question (F) of the symptom questionnaire is a compilation of the symptom questions used in the IC overlay test and assesses state, rather than trait. The questions ask about task-specific questions. Since these are the questions that practitioners using the IS believe are indicative of VS [13], it is pragmatic to use these symptoms as a primary outcome measure. These sections of the questionnaire comprised 6 questions, arranged on a five-point scale for sections (D) and (E) and a ten-point scale for (F) (Fig. 3).

The specific measurement variable will be symptom scores on a 5- and 10-point quantitative scale, representing symptom severity, with pair A versus pair B. However, a pilot study of the ARUTIS questionnaire will be conducted before finalisation to ensure that all items are age-appropriate and relevant and to verify that the questionnaire does not impose an undue burden on trial participants.

#### Secondary outcome measures

Secondary outcome measures will assess academic activity and behaviour by analysing surveys completed by parents/guardians and teachers under the two different intervention conditions.

Children with poor reading skills often have a heightened sensitivity to their poor reading and can be embarrassed by their perceived failures to read well. This can sometimes result in disaffection with reading as a whole and may contribute to poor academic outcomes and challenging behaviour. Studies have shown that 24% to 52% of children with learning difficulties present with problems with their behaviour [34]. Reading is involved in most schoolwork, and it seems likely that if a student has symptoms of headache, eyestrain, and visual perceptual distortions when reading, then this would deter them from reading [35]. This would be increasingly problematic as the child progresses through school because there will be a decrease in font size [36] and spacing which is likely to be associated with increased symptoms of VS.

Children suffering from symptoms of VS may manifest these as changes in behaviour (e.g. to avoid reading) rather than vocalising the symptoms of VS. Therefore, we have included an academic behaviour survey (seen in Additional file 6), to be completed by parents/guardians and a teacher at the end of each month of wear.

The specific measurement variable will be scored on a 5-point quantitative scale, representing the severity of VS symptoms, which manifest academically and behaviourally in pair A versus pair B. The analysis will be carried out at the end of the 3-month trial, as participants will complete questionnaires at baseline (referring to the situation before interventions) and at the end of each wearing period (referring to the situation with each intervention). The method of aggregation will be to average scores, and analysis of the questionnaire, along with the results from the primary outcome measure, will reveal whether pair A or pair B created the greatest reduction in VS symptoms.

Academic survey results may be influenced by factors unrelated to VS. However, the surveys are to be completed both before and after the intervention. The post-intervention results will reflect if VS symptoms create any effect on academic performance, rather than being the sole reason for poor academic achievement.

#### Tertiary outcome measures

The tertiary outcome measure will assess reading speed using the WRRT. The WRRT will be measured before prescribing PTLs, with each pair of PTLs, and at the end of the clinical trial in phase 5, as described above. To reduce variability and assess reliability, the test will be administered twice in each condition (with or without tints). The testing order will be ABBA, with A randomly allocated to one of the conditions.

Previous studies have shown that for participants with VS, PTLs and IOs increase reading speed [25, 37] measured on the WRRT. Conventional reading tests [38] are designed for educational use to assess long-term improvement and are commonly used to estimate reading age. These educational tests often use large print that is visually unchallenging and are not suitable for assessing the immediate short-term effect of optometric interventions.

In contrast, the WRRT was designed not for educational use but to assess the immediate effect of optometric interventions [25, 37] and comprises four passages, each taking 1 min to administer. This approach is highly relevant in measuring the effect of PTLs. Research reveals that the WRRT is a valid measurement of within-subject treatment effects and, therefore, well-suited to the research [25].

The specific measurement variable will be scored on a quantitative scale representing the number of words read correctly in 1 min on the WRRT with pair A vs pair B. The analysis will be carried out at the end of the 3-month trial, as participants will complete the WRRT throughout the trial, administered at baseline (referring to the situation before interventions) and at the end of each wearing period (referring to the situation with each intervention). The method of aggregation will be to compare the number of words read and will reveal whether pair A or pair B created the greatest reduction in VS symptoms.

### Participant timeline

See Table 2.

**Table 2:**
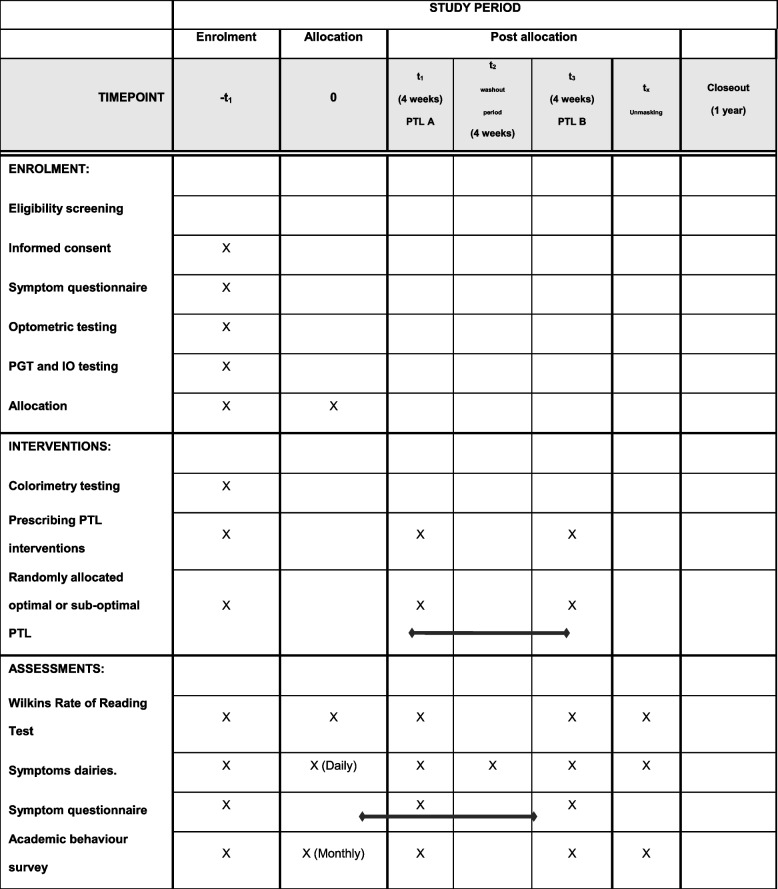
Participant timeline using the SPRIT template

### Sample size

A sample size ($n$ = 120) was determined based on the sample size calculation for AB/BA crossover trials described by Jones and Kenward [39]:

$$n=\frac{2{({Z}_{1-\beta }+{Z}_{1-\alpha /2})}^{2} {\sigma }_{\omega }^{2}}{{\mu }_{d}^{2}}$$

where:

- $n$ = total needed sample size
- $({Z}_{1-\beta }+{Z}_{1-\alpha /2})$ = sum of $z$-scores at which the tails of the normal distribution correspond to probabilities $\alpha$ and $\beta$, where $\alpha$ is the desired significance level for hypothesis testing, and $1-\beta$ is the desired power of the test
- $\sigma$ = within-subjects standard deviation of symptom scores
- ${\mu }_{d}$ = mean difference in scores between control and experimental conditions $({\mu }_{ctrl}- {\mu }_{expt})$

This total sample size estimate was calculated using an effect size of half a standard deviation (0.5 SD), 90% power, a 0.05 probability of a type I error ($n$ = 88), and an allowance for individuals who might withdraw from the study.

To establish whether an experimental treatment is superior to the control, the convention is to set the significance level (*α*) at 0.05, as recommended by Julious [40]. Furthermore, a statistical power of 0.90 (*β*= 0.10) was selected, as this is the recommended standard for crossover trials [39, 40].

When discussing the effect size for this RCT, it was apparent that there are no prior data to aid the selection of effect size, and this RCT is proposing a new outcome measure. Therefore, it was necessary to investigate existing statistical rationales to assist with this decision. An effect size (standardised mean difference) of 0.5 SD was chosen based on Lakens [41] and Norman et al. [41, 42].

### Recruitment

It is anticipated that most participants will be recruited from the Anglia Ruskin University (ARU) eye clinic by informing self-referred VS participants of this RCT. This clinic has offered specialist services to people with suspected VS for over 10 years and has a reputation for excellence in this field [35]. This will be the initial approach, but as recommended by Loudon et al., if this does not produce the required number, additional steps will be taken [43]. Specifically, publicity will be generated by advertisements placed within the ARU, the ARU eye clinic (including the orthoptist department), and local optical practices with the permission of practice managers and/or owners.

Cash payments will not be made, but PTLs will be provided free of charge for participants of the RCT. A basic spectacle frame will be offered free of charge, with participants able to use a more expensive frame if they fund this themselves. The provision of reduced price/free precision tinted spectacles means that some participants may be included who would not otherwise have been able to afford precision tinted spectacles. Data collection and any optometric testing will only begin after informed consent has been given.

## Assignment of interventions: allocation

### Sequence generation

The sequence generation will use block randomisation, with blocks of 4, from www.sealedenvelope.com. The use of blocks of 4 means that as long as the number of participants is a multiple of 4, there will be an equal number of participants wearing each intervention in the first period (balanced design).

Participants will be told that 50% of participants will receive the optimal colour first and 50% will receive a slightly different colour first. Each participant will swap to the other colour after 1 month of wear.

When the specification of both tints has been determined at the colorimetry assessment, the participant will choose a spectacle frame in which either the optimal or sub-optimal tint will be glazed. A technician (not the research optometrist) will check the frames, lens optical properties, and tint. Each participant will be provided with one spectacle frame which will be reglazed to the other tint at crossover.

The researcher, participants, parents, teachers, etc., will be masked as to whether each participant receives the optimal or sub-optimal tint first. This is possible because during colorimetry, when each colour is selected, colours are changed slowly so that the person being tested adapts to the colour and therefore is unlikely to be aware of the difference in colour appearance of the optimal and sub-optimal tint. Further, participants will receive the first pair of spectacles some weeks after colorimetry, so they will be likely to have forgotten their detailed perceptions during colorimetry.

### Implementation

Participants will be given participant numbers in sequence. The lead researcher (ZR) will undertake colorimetry and will send the results for each participant to AW. For each participant, AW will provide NT with the PTL specification for the optimal and sub-optimal tints. BE will provide a list of randomisation codes to NT, specifying for each participant number whether the first pair should be the optimal or sub-optimal colour. NT will organise the ordering of the PTL with the dispensing team at Cerium. ZR will conduct all participants’ testing and will be unaware of whether the optimal tint is worn first or second.

### Who will be blinded

The lead researcher, trial participants, parents/guardians, teachers, and all members of the research team involved in the statistical analysis will be masked. During the statistical analyses, tints will be referred to as A and B and the code, identifying A or B as the optimal/sub-optimal, will only be broken once the statistical analysis is completed.

### Plans for assessment and collection of outcomes

Please see Additional file 3.

### Data management

Please see Additional file 3.

### Confidentiality

Data from the optometric testing and visual stress testing will be kept safe within the university eye clinic as per GDPR, and the records will not be removed from the university clinic setting. The research data, which includes colorimetry data of participants of the crossover trial, will be fully anonymised, and data files for analysis by members of the research team will, in addition to anonymisation, be password protected.

### Plans for collection, laboratory evaluation, and storage of biological specimens for genetic or molecular analysis in this trial/future use

Not applicable, there will be no use of biological specimens in this study.

## Statistical methods

### Statistical methods for primary and secondary outcomes

To assess the efficacy of PTLs, intervention effects will be compared by analysing symptoms between pairs A and B. A pre-specified outcome hierarchy has been implemented in this RCT. The primary outcome is the change in symptoms between optimal and sub-optimal PTLs, assessing separately (1) immediately recalled (“live”) symptoms with daily diaries and (2) recollected symptoms, using the ARUTIS Symptom Questionnaire. Secondary outcomes include parent and teacher Academic Behaviour Survey score aggregates. The analysis will be conducted at the conclusion of the 3-month trial.

Descriptive analysis will initially summarise the data by converting the ARUTIS Symptom Questionnaire and Daily Symptom Diary scores into numerical values. The aggregation method will combine symptom scores within the end-of-period questionnaire to produce a single score, as shown in Table 3. Daily symptom scores from the symptom questionnaire will also be converted to numerical values and calculated as the average across days with symptoms.

**Table 3:** Aggregation of scores for the ARUTIS Symptom Questionnaire

Component of questionnaire	Number of questions	Scale (max per question)	Maximum score
B	1	1–10	10
D	6	0–5	30
E	1	0–5	5
F	5	0–9	50
Total aggregate symptom score	–	–	95

Since the primary outcome, symptoms, is assessed in two ways (daily diary score and end-of-period questionnaire), considerations were made as to whether a Bonferroni correction is necessary. However, these two methods evaluate symptoms in fundamentally different ways. The daily diaries offer near real-time estimates of symptoms, providing a “live” assessment. In contrast, the questionnaires used at the end of each wearing period give a more long-term recollection of symptoms during that time, especially when considering a standardised passage of text through the relevant intervention. As the two approaches assess different hypotheses regarding symptoms, it is believed that a Bonferroni correction is not required. However, for each method, one aggregate score should be obtained.

Secondary outcomes include parent and teacher Academic Behaviour Survey score aggregates, and the tertiary outcome measure will assess reading speed using the WRRT under the two intervention conditions.

Initially, all data will be checked by scatter plots of raw data. All data will be tested for normality using the Shapiro-Wilk test, and non-parametric data will be transformed to parametric where possible. Simple comparisons will use paired *t*-tests, or the Wilcoxon signed-rank test as appropriate. For more complex analyses, a Mixed-Effects Model for Repeated Measures (MMRM) will be carried out to account for the crossover design, including fixed effects (intervention type, period, and sequence) and random effects (participant variability).

A confirmatory check will be performed to test for order effects (e.g. whether participants who have the optimal tint after the sub-optimal tint have a statistically different outcome from those who have the sub-optimal tint after the optimal tint). If such an effect is found, key analyses will be repeated whilst controlling for the order effect. In addition, an analysis of the effects of the first tint alone will be compared across participants. The analysis will have low power and will be confirmatory, but it is independent of order effects.

Participants for whom there is a complete set of symptom diary data for at least 2 weeks of wear with both interventions will be analysed on an intention-to-treat basis (ITT) [44]. In the unlikely event that any participants are misallocated, then key analyses will also be run according to a per protocol approach. For the symptom questionnaire conducted before enrolment in the trial and for the academic behaviour surveys, data will only be analysed for participants for whom data are available for *both*interventions [44].

Sensitivity analysis will also be carried out to confirm the robustness of the data produced.

The secondary outcome survey measures will be converted to numeric values. Scores will be aggregated and averaged across surveys and then prepared for statistical analysis using the approaches described above. Tertiary outcome measures will be assessed separately as an exploratory analysis. Tertiary outcome measures will undergo reliability testing using the intraclass correlation coefficient, and analysis for order effects using repeated measures ANOVA, before comparing interventions using standard *t*-tests.

All statistical analyses will be conducted using R (version 4.3.1, R Core Team 2023).

### Interim analyses

There will not be access to interim results.

### Plans to give access to the full protocol, participant-level data, and statistical code

After data analysis, the de-identified results will be placed in a public repository for access by other interested researchers.

### Composition of the coordinating centre and trial steering committee

The first five co-authors of this manuscript will fulfil the roles of a trial steering committee. The lead researcher (ZR) will provide organisational support, meeting with other members of the research team monthly to ensure that the study is well-conducted.

### Composition of the data monitoring committee, its role and reporting structure

The intervention under investigation (tinted lenses) is safe, and the sub-optimal colour, although not the optimal colour, will be chosen as a similar colour that is unlikely to cause significant symptoms compared with no tints. However, as a precaution, all participants will be told, in writing (in the PIS) and verbally, that they can discontinue wear at any time. Given this precaution and the safe nature of the intervention, it is thought that a data monitoring committee is not needed. The interventions are safe (PTLs), and participants will be informed (verbally and in writing) that they can stop wearing the coloured lenses at any time.

### Adverse event reporting and harms

The risks of harm are very low, and there is no ophthalmic drug intervention. Full optometric testing, which is a key part of this clinical trial, involves tests widely used in routine optometric practice. PTLs are designed to reduce symptoms of visual stress and improve reading speed. However, in the unlikely event that there is a marked increase in symptoms or reduced reading performance, affecting behaviour or academic outcomes, the participants can stop wearing the PTL and drop out of the trial. There have been no studies showing this effect.

### Frequency and plans for auditing trial conduct

This work is a doctorate project for ZR. The on-campus supervisory team, led by PA, will audit the research records for approximately every fifth participant.

### Plans for communicating important protocol amendments to relevant parties (e.g. trial participants, ethical committees)

Protocol amendments will be discussed within the research team, and if it is agreed they are essential, approval will be requested from the ethics committee. The trial registry will be updated.

### Dissemination plans

The study protocol has been presented at an international conference (European Academy of Optometry and Optics, 2022) and is being published herewith. The results will be presented at one or more international conference(s) and published in one or more peer-reviewed journal article(s). Any participants who express an interest in the results will be invited to email ZR who will send a de-identified summary of the main findings in lay language.

## Discussion

A protocol is outlined for a double-masked, randomised, controlled, crossover clinical trial (RCT) involving young people aged 9–18 years who meet contemporary diagnostic criteria for VS. The trial will investigate whether PTLs prescribed using the IS are beneficial. Systematic reviews [13, 20] in 2016 criticised the lack of consistent diagnostic criteria and in response criteria were published in 2017 [2]. This clinical trial will be the first study of children to adopt these diagnostic criteria. Both major reviews on this topic agree that more clinical trials are required to establish whether PTLs confer a clinical benefit in the treatment of VS.

A strength of this study is the decision to use symptoms as the primary outcome measure, which is strongly justified by the literature, and the development of a symptom questionnaire based on the literature to identify and quantify symptoms of VS. VS is a construct that is not observable or measurable directly; in statistical terms, it is a latent variable that is accessible only through certain measurable indicator variables, such as the frequency/severity of symptoms. Therefore, to support the idea that the overarching construct is valid, there was a requirement for a symptom questionnaire to be created with several VS indicators from published research [2, 10, 13]. A symptom questionnaire for VS has recently been developed for adults [45], but none is available for children and hence the development of the approach described in Methods.

A notable strength of this study is its pragmatic design, which allows data collection to occur across different phases of the school academic year, depending on the date of participant enrolment, as the study extends over 3 months. This introduces natural variation in academic and visual demands, creating differences in PTL usage and reflecting real-world use of PTLs. It captures the potential benefits of PTLs in managing VS symptoms during study-related tasks and daily visual activities. This design enhances the generalisability and real-life relevance of the results. For practical reasons, all participants will not start at the same time, but rather, enrolment in the study will be staggered over many months. Therefore, randomisation of tint allocation to wear period will average out any effects of holidays.

Another strength of this study is the potential impact on society. If an individual suffers from symptoms of visual distortion, headaches, and eyestrain when reading, there is likely to be a tendency to avoid reading which may impact their educational achievement. This RCT will address the core symptoms of VS and help identify whether PTL have an effect on symptoms and WRRT performance in sufferers of VS using the Intuitive System. Improving the understanding of symptoms and potential treatment in those who report the symptoms of VS can provide health care professionals such as optometrists (who will be the most likely to encounter such symptoms in routine eye examinations) to provide patients with advice and, if appropriate, treatment.

Limitations of the trial are as follows. First, there is a risk with a single-site trial within a specialist clinic that the results may not be directly applicable to a more general population. However, the trial will recruit school-age participants from a variety of schools. Additionally, VS is typically treated in specialist optometric clinics, so the choice to base the trial in such a clinic is pragmatic as it reflects typical clinical practice.

Second, due to the requirement for daily diaries and multiple visits, there is a risk of participant attrition. However, the lead researcher aims to develop a positive rapport with participants and send frequent reminders as explained above. The importance of daily diaries lies within the findings of the original 1994 trial [9], where the daily diary analysis produced findings in the original RCT that were statistically significant, justifying a similar approach in this study. The use of reminders by modern technologies (e.g. SMS, WhatsApp, emails) should improve compliance with diary completion compared with the original study.

The results of this RCT aim to strengthen the evidence base concerning the existence and, if appropriate, treatment of VS using the IS.

## Trial status

Ethical approval has been granted by Anglia Ruskin University's ethics. The protocol has been registered at ClinicalTrials.gov https://clinicaltrials.gov/study/NCT06093516?cond=visual%20stress&rank=1.

Protocol version: 20th December 2024 V2.5.

Recruitment has not begun.

Estimated start date: 1st February 2026.

Estimated end date: 1st July 2026.

## Supplementary Information

Additional file 1.

Additional file 2.

Additional file 3.

Additional file 4.

Additional file 5.

Additional file 6.

Additional file 7.

Additional file 8.

Additional file 9.

Additional file 10.

Additional file 11.

## Data Availability

At the completion of the analysis by the study team, the de-identified data will be placed in a public data repository.

## Abbreviations

RCT: Randomised controlled trial
VS: Visual stress
PLTs: Precision tinted lenses
TLs: Tinted lenses
WRRT: Wilkins Rate of Reading Test
IS: Intuitive System
IC: Intuitive Colorimeter
IO: Intuitive Overlays
PGT: Pattern Glare Test
ARU: Anglia Ruskin University
