# Supplementary material for: The ARUTIS Study (Anglia Ruskin University Trial of the Intuitive System): a single-centre, double-masked randomised controlled crossover trial of precision tinted lenses for visual stress: study protocol for a randomised controlled trial
Source: Trials. 2025 Dec 16;27:61. doi: 10.1186/s13063-025-09305-8 (PMC12822186; doi:10.1186/s13063-025-09305-8)
Supplement: Supplementary file 1 — Additional file 1. [file 13063_2025_9305_MOESM1_ESM.docx]

| **Parameter** | **Test method** | **Criterion** | **Action if fails criterion** |
| --- | --- | --- | --- |
| Ocular pathology | Biomicroscopy & ophthalmoscopy (with dilation if required). | Ocular pathology requiring referral or likely to interfere with vision. | Exclude (and refer as appropriate following College of Optometrists guidelines). |
| Refractive error | Non-cycloplegic retinoscopy & subjective; cycloplegic if required.^1^ | Prescribe refractive errors: that are clinically significant for age and where correction likely to improve visual development and function.^2^ | If first spectacles are required or existing spectacles need to be updated, postpone entry until new spectacles are worn for 1 month. |
| Visual acuity | LogMAR (letter-by-letter scoring) | Habitual binocular visual acuity (VA) worse than 0.2 LogMAR with spectacle correction. | Postpone entry until VA at least 0.2 LogMAR with spectacle correction. |
| Ocular motility | Pen torch in cardinal positions of gaze. | Nystagmus or incomitancy. | Exclude. |
| Manifest strabismus | Cover test. | Recent onset or unstable. | Postpone entry if requires referral/treatment until stable for 3 months. |
| Decompensated heterophoria/binocular instability | Cover test, Mallett fixation disparity test, fusional reserves. | Abnormal by Table 5.2 in Evans.^1^ | Postpone entry until treatment or correction has normalised for >1 month. |
| Accommodation | RAF rule.  MEM retinoscopy. | Below Hofstetter minimum^1^ using formula below.  Minimum amplitude (D)=15.0−(0.25×age in years) | Postpone entry until treatment or correction^1^has normalised for >1 month. |

Additional File 1

Supplementary Table 1

Optometric testing and exclusion criteria

The tests of ocular alignment and accommodation will be carried out with participants wearing any spectacles that they wear habitually (>50% of the time) when studying. Colour vision defects will not be an exclusion criterion.
Participants with colour vision deficiencies and any neurodiverse participants, such as those with dyslexia, dyspraxia, or ADHD, will be permitted. However, enrolment of participants will be postponed for three months if medication has been recently initiated or adjusted, to minimise confounding effects on vision caused by medication.

References

1. Evans Bruce JW. Pickwell’s Binocular Vision Anomalies. Sixth ed. Philadelphia: Elsevier; 2022.

2. Leat SJ. To prescribe or not to prescribe? Guidelines for spectacle prescribing in infants and children. Clin Exp Optom. 2011;94(6):514–27.
