# Supplementary material for: The ARUTIS Study (Anglia Ruskin University Trial of the Intuitive System): a single-centre, double-masked randomised controlled crossover trial of precision tinted lenses for visual stress: study protocol for a randomised controlled trial
Source: Trials. 2025 Dec 16;27:61. doi: 10.1186/s13063-025-09305-8 (PMC12822186; doi:10.1186/s13063-025-09305-8)
Supplement: Supplementary file 2 — Additional file 2. [file 13063_2025_9305_MOESM2_ESM.docx]

Additional file 2

Detailed procedure of participant flow of RCT

**Stage 1**

There will be three sections to the recruitment process.

**Stage 1a**

Participants will be recruited from the Anglia Ruskin University (ARU) eye clinic, by informing self-referred VS patients of this RCT. This clinic has offered specialist services to people with suspected VS for over 10 years and has a reputation for excellence in this field.

PTLs will be provided free of charge for participants of the RCT. A basic spectacle frame will be offered free of charge, with participants able to use a more expensive frame if they fund this themselves. The provision of reduced price/free precision tinted spectacles means that some participants may be included who would not have been able to afford PTLs.

Initial publicity will also be generated by adverts placed within ARU, the ARU eye clinic (including the orthoptist department), and local optical practices with permission only.

### **Stage 1b**

Data collection will only begin after informed consent has been given. A symptom questionnaire will take place (see Additional file 4) and optometric testing summarized in Supplementary Table 1; Additional File 1 will take place. Participants will be given a 10-minute break.

**Stage 1c**

Participants will be individually tested by the lead researcher with the PGT and IO. Participants who report that there is no single overlay (or pairwise combination)^1^ that is helpful will be thanked for their help and exited from the recruitment process and given advice or a referral for their symptoms within the College of Optometrists guidelines by the lead researcher.

Participants who report that an overlay improves their symptoms will be tested with and without the overlay with the WRRT.

Participants who show an immediate >15% improvement in the WRRT with IO and pass the PGT criterion (Table 1) can continue onto the RCT.

Participants who do not pass the PGT or WRRT criteria will be exited from the recruitment process and given advice or a referral for their symptoms within the College of Optometrists guidelines by the lead researcher.

Participants who pass the PGT criterion but not the WRRT criterion or vice versa will be issued with an IO or IOs of their preferred colour to try, if they wish, as and when they find them helpful.

These participants will be contacted after at least three months and asked if they have found their overlay(s) helpful and if they are still using them. If they are no longer using their overlay they will be exited from the recruitment process, with the recommendation that they consult an optometrist about their symptoms. If they are still using the overlay and find it helpful, then they will be admitted to the RCT.

**Stage 2**
Participants will be randomly allocated (with masking) to wear either the optimal (active) colour or sub-optimal (control) colour first. Colorimetry testing is as follows:

After the optometric testing, the participant will be given a break for at least 10 minutes and then the colorimetry testing will be undertaken. Colorimetry will be carried out with participants wearing any spectacles they usually wear when reading, with the following procedure (an associate researcher will also assist testing, to facilitate masking of the lead researcher and the research team):

1. The participant is asked to describe any perceptual distortion and discomfort of the text in the colorimeter. Their description is used.
2. At each of the 12 hues in turn the saturation of colour is increased from white to modest saturation (30) and after 5s returned to white. The participant is asked to compare the coloured text with the white and report any differences in the distortion/discomfort.
3. At the best of the 12 hues, the participant adjusts the saturation to optimise the clarity and comfort of the text (comfort is more important than clarity).
4. At this saturation, the hue is adjusted by small amounts and re-optimised. The saturation is then minimised.
5. At the re-optimised hue/saturation, the brightness is reduced to assess its effects on comfort. If the lower brightness is preferred the saturation is increased slightly to see if the high brightness is better tolerated.
6. An associate researcher (to facilitate masking) will make up a combination of trial lenses to match the setting without letting the participant see them and will note the combination required. The associate researcher will also perform the WRRT after each tint is found, to maintain the masking element of this RCT.
7. Participants who do not give consistent results will be excluded.

After colorimetry, participants will select a spectacle frame. Two pairs of PTLs will be created by the manufacturing opticians (at Cerium Visual Technologies), one featuring the optimal colour and the other a slightly sub-optimal colour. The sub-optimal colour will be chosen as a colour with similar saturation to the optimal but differing in u’v’ colour space by 0.07. Two colours, equidistant from the optimal in terms of radial distance, will be considered. The colour that shares the name of the optimal colour or appears most similar will be selected. The 0.07 distance is chosen to be comparable to the previous RCT^1^ and remains stable across common variations in lighting conditions.^1^

Participants will then receive two academic behaviour surveys^2,3^ (see additional file 6). These surveys will serve as baseline assessments for academic behaviour and will be accompanied by stamp-addressed envelopes for the parent and teacher to complete and return.

When both tint specifications have been determined, the participant will choose a desired frame of choice in which either the optimal or sub-optimal tint will be glazed in.

There will be a 50:50 allocation ratio of the tint worn first to detect superiority. The sequence generation will use Block Randomisation, with blocks of 4, from [www.sealedenvelope.com](http://www.sealedenvelope.com). The use of blocks of 4 means that as long as the number of participants is a multiple of 4, there will be an equal number of participants starting with optimal/suboptimal tint (balanced design). The associate researcher will store this information in a password-protected spreadsheet to which only they have access and send it. Information on which pair (first or second) is the optimal colour will be placed in a sealed envelope, labelled with the participant’s name, for use as specified below. The frames will be sent to Glaze.

We will have one frame and reglaze to the other tint at cross-over. The researcher, participants, parents, teachers, etc will be masked as to whether each participant receives the optimal or sub-optimal tint first. Participants will be given a symptom diary to fill out every day until the collection appointment of PTL, this will be for approximately 2 weeks.

**Stage 3**The short-term effects of the first pair of PTLs will be analysed.

At collection, the associate researcher (not the lead researcher) will check the frames, lens optical properties, and tint.

- 1. At the collection appointment for the first pair of PTLs, the frames will be fitted to the participant and the participant will be asked if they are content to wear the PTLs for studying at school and at home. If the participant is not content to wear the spectacles the participant will be continued in the study, allowing them to try the second pair. In this situation, data will still be gathered (e.g., diary), but will not be included in the analysis. Once the frames with the PTLs have been fitted to the participant, the Wilkins Rate of Reading Test (WRRT) will be carried out with and without the PTLs. The tests will be given in the order ABBA with a random allocation of the PTL to A or B. If there is an optical prescription in the PTLs then the comparison will be between the habitual (un-tinted) spectacles and the PTLs held in front of spectacles or trial frames with the appropriate prescription.

     Before the participant leaves the collection appointment, they will receive two academic behaviour surveys (see additional file 6). These surveys will need to be completed after one month of wear. The surveys will be accompanied by stamp-addressed envelopes for both the parent and teacher to complete and return.
  2. Throughout the first month of spectacle wear, participants will be asked to complete a daily diary of their symptoms and to estimate for how many hours they wore the PTLs. They will be reminded weekly by phone, and every third day by text or email. Participants will be told that they can discontinue wearing their PTLs if they find them uncomfortable, but if the frames need adjustment this will be arranged. An online diary will be used so that data is saved to the researcher's database every day, but paper versions will also be available for any participants who do not have computer access. It is anticipated all or nearly all participants will use the online diary, which will be monitored so that participants who forget can receive additional reminders.
  3. At the end of the first wearing period (one month), participants will receive a symptom questionnaire to log their symptoms with PTL and a text will be sent out to remind participants to complete post the academic behaviour surveys back to the research team.
  4. Participants will then be contacted and asked to return their first pair of spectacles in prepaid packaging. This is to give the participant time to forget about the exact colour of the first PTLs.

Participants will be told to continue their symptom diaries throughout the one-month washout phase.

The washout period of one month will then begin, after which they will reattend the research clinic to collect the second pair.

**Stage 4**
After a washout period of one month, participants will crossover to the other pair of PTLs. The short-term effects from the second pair of PTLs will be assessed. Part a, b and c as above is repeated.

**Stage 5**
After both pairs have been worn, the participant will return for another visit:

- 1. At the end of the one month with the second pair of PTLs, participants will be seen again in the research clinic. If they have a refractive correction, they will be asked to bring with them their untinted spectacles. The WRRT will be undertaken, comparing the first pair of PTLs with the second pair of PTLs. The PTL trial frame will be used to present both PTL specifications, so that the same mode of presentation will be used for both tints. The usual ABBA sequence will be used, and the first tint will be randomly allocated to A or B.
  2. After the WRRT scores have been entered into the database of results, the lead researcher views (but not edit) the diary data and these and the WRRT data will be considered, to decide with the participant, about which pair they wish to keep.

Before data analysis, a member of staff at the University who is not involved in the research will break the masked code, discovering for each participant whether the first or second pair of tints was the optimal colour. This will not be revealed to the researchers, and instead of optimal/suboptimal, this third party will apply labels such as alpha/beta.

Only this third party will know whether, for each participant, alpha is the optimal or suboptimal colour. Therefore, the Lead Researcher can carry out data analysis and discuss it with the rest of the research team whilst the team remains masked. Once the analysis is complete, the third party will reveal whether alpha or beta is the optimal tint specification.

While patient preference is important, in this study, participants will be prescribed based on the tint that produces the fewe**st symptoms.** The RCT is designed to **establish causality** by measuring changes in symptoms between the optimal and suboptimal PTL, rather than relying on preference. Assessing preference could compromise masking and introduce bias, particularly given the **age range of participants,** who may be influenced by colour appeal or suggestion

References

1. Wilkins A, Sihra N, Smith IN. How precise do precision tints have to be and how many are necessary? Ophthalmic and Physiological Optics. 2005 May;25(3):269–76.

2. Rouse M, Borsting E, Mitchell GL, Kulp MT, Scheiman M, Amster D, et al. Academic behaviors in children with convergence insufficiency with and without parent-reported ADHD. Optometry and Vision Science. 2009 Oct;86(10):1169–77.

3. Borsting E, Lynn Mitchell G, Kulp MT, Scheiman M, Amster DM, Cotter S, et al. Improvement in Academic Behaviors After Successful Treatment of Convergence Insufficiency. Journal Of American Academy of Optometry. 2012;89(1):12–7.
