# Supplementary material for: The ARUTIS Study (Anglia Ruskin University Trial of the Intuitive System): a single-centre, double-masked randomised controlled crossover trial of precision tinted lenses for visual stress: study protocol for a randomised controlled trial
Source: Trials. 2025 Dec 16;27:61. doi: 10.1186/s13063-025-09305-8 (PMC12822186; doi:10.1186/s13063-025-09305-8)
Supplement: Supplementary file 3 — Additional file 3. [file 13063_2025_9305_MOESM3_ESM.docx]

Additional file 3

Data collection and management

Since participants will be recruited from the university eye clinics and some will seek continued eye care in these clinics, there is a need to differentiate between **clinical data** that would ordinarily be gathered in these clinics and will need to be accessible to other staff in the clinic and **research data** that is not required for future clinical care and will be used wholly and exclusively for research. At recruitment, each participant will be assigned a participant code. This code will be used to ensure they remain anonymous in data analysis.

Initial clinical data from the eye examination, visual stress testing and colorimetry data will be collected by the lead researcher (ZR), and results will be recorded with standard clinic record cards. These clinical data will, in the usual way, be kept securely at the eye clinic for 10 years following the guidelines of the College of Optometrists. For study participants, copies of these data will be stored in the research data, with participants only identified by their participant code and with no personal data (age will be stored in years and months so that date of birth will not be included in research records).

All data required for the randomised controlled crossover trial (such as participant code, age, prescription, and colorimetry results) will be entered and stored electronically via an Excel spreadsheet with a password entry. This data entry will be done at the ARU eye clinic. Keeping results from the clinical trial stored electronically will reduce the time required upon analysing data after the clinical trial has ended. This main data spreadsheet used for the clinical trial will not contain any personal data and will only identify participants by their participant code.

Date of birth will not be recorded; age will be recorded in years and months. There will be a separate personal data spreadsheet, that is password controlled and uses a different password to the main data spreadsheet (also kept at ARU eye clinic). This personal data spreadsheet will be kept by the Lead Researcher and will cross-reference the participant code with personal data (name, address, telephone number, and email; for contacting the participant).

Symptom questionnaires and diaries will be created on Online Surveys, which is a survey builder recommended by Anglia Ruskin University and is GDPR compliant. The data collected from the questionnaire will be password protected; no researcher or collaborator will have access to this until the unmasking stage of the clinical trial.

Once participants have completed colorimetry testing, participants will receive a link to a symptom diary with instructions to complete every day until the end of the clinical trial. Data from these diaries will be exported to the same Excel spreadsheet at the end of the trial which contains the colorimetry results next to participant code. Results from the academic behaviour survey will also be exported to the same Excel spreadsheet. No researcher or collaborator will have access to this until the unmasking stage of the clinical trial. All research records will be anonymised and stored in a secure place and manner within the university for 3 years after completion of the study.
