# Supplementary material for: The ARUTIS Study (Anglia Ruskin University Trial of the Intuitive System): a single-centre, double-masked randomised controlled crossover trial of precision tinted lenses for visual stress: study protocol for a randomised controlled trial
Source: Trials. 2025 Dec 16;27:61. doi: 10.1186/s13063-025-09305-8 (PMC12822186; doi:10.1186/s13063-025-09305-8)

## ARUTIS (Anglia Ruskin University Trial of the Intuitive System) Symptom questionnaire

1. Please indicate in a few words any problems you experience when reading a passage of text, for example, in a book:
2. Please estimate how bad (severe) this problem is, or these problems are, by writing a number from 1 (hardly noticeable) to 10 (very bad, interferes with reading all the time):
3. What is the longest period you spent reading last week (minutes or hours):

P(Please say if minutes/hours)

1. When reading a passage of text in a book, how often do you experience the following? For each question, please tick the box that applies:

|  | Never  when I read | Rarely  when I read | Sometimes when I read | Often  when I read | Always  when I read |
| --- | --- | --- | --- | --- | --- |
| How often do words move? |  |  |  |  |  |
| How often do words merge? |  |  |  |  |  |
| How often do you see shadows or patterns in text? |  |  |  |  |  |
| How often does text stand out in 3-D? |  |  |  |  |  |
| How often do letters fade or darken? |  |  |  |  |  |
| How often do you experience headaches from reading? |  |  |  |  |  |

1. How often do you experience the problem below at any time (not just when reading)? Please tick the box that applies:

|  | Never | Rarely | Sometimes | Often | Always |
| --- | --- | --- | --- | --- | --- |
| How often do you experience discomfort with some artificial lights or flicker? |  |  |  |  |  |

1. Please look at the passage of text on the next page and then describe whether you see the problems in the table below. For each issue, please estimate how bad (severe) this problem is by writing a number from 0 (does not occur) to 9 (very bad):

|  | Score  (0-9) |
| --- | --- |
| Do the letters move? |  |
| Are the letters fuzzy (blurred)? |  |
| Are the words too close together? |  |
| Does the page look too bright? |  |
| Does it hurt your eyes to look at the page? |  |


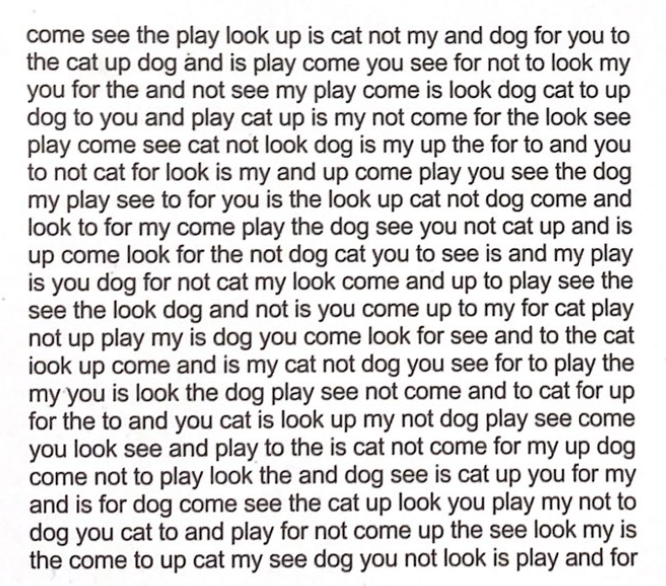

Supplement: Supplementary file 4 — Additional file 4. [file 13063_2025_9305_MOESM4_ESM.docx]
