# Supplementary material for: The ARUTIS Study (Anglia Ruskin University Trial of the Intuitive System): a single-centre, double-masked randomised controlled crossover trial of precision tinted lenses for visual stress: study protocol for a randomised controlled trial
Source: Trials. 2025 Dec 16;27:61. doi: 10.1186/s13063-025-09305-8 (PMC12822186; doi:10.1186/s13063-025-09305-8)
Supplement: Supplementary file 5 — Additional file 5. [file 13063_2025_9305_MOESM5_ESM.docx]

**Daily diary**

Name .........................................................

Week beginning Monday.....................

*Every day, please tick the box or boxes that apply, write down the number of hours you wore your glasses, and complete the appropriate record sheets, if necessary.*

Monday

I wore my coloured glasses for .............hours.

I did not have any symptoms €

I had a headache from reading €

I had eyestrain (or eye discomfort) €

Tuesday

I wore my coloured glasses for .............hours.

I did not have any symptoms €

I had a headache from reading €

I had eyestrain (or eye discomfort) €

Wednesday

I wore my coloured glasses for .............hours.

I did not have any symptoms €

I had a headache from reading €

I had eyestrain (or eye discomfort) €

Thursday

I wore my coloured glasses for .............hours.

I did not have any symptoms €

I had a headache from reading €

I had eyestrain (or eye discomfort) €

Friday

I wore my coloured glasses for .............hours.

I did not have any symptoms €

I had a headache from reading €

I had eyestrain (or eye discomfort) €

Saturday

I wore my coloured glasses for .............hours.

I did not have any symptoms €

I had a headache from reading €

I had eyestrain (or eye discomfort) €

Sunday

I wore my coloured glasses for .............hours.

I did not have any symptoms €

I had a headache from reading €

I had eyestrain (or eye discomfort) €
