# Supplementary material for: The ARUTIS Study (Anglia Ruskin University Trial of the Intuitive System): a single-centre, double-masked randomised controlled crossover trial of precision tinted lenses for visual stress: study protocol for a randomised controlled trial
Source: Trials. 2025 Dec 16;27:61. doi: 10.1186/s13063-025-09305-8 (PMC12822186; doi:10.1186/s13063-025-09305-8)
Supplement: Supplementary file 6 — Additional file 6. [file 13063_2025_9305_MOESM6_ESM.docx]

Teacher Academic Behaviour Survey

Survey: 1/2/3

Date:

Participant Name:

|  |  | **Never** | **Rarely** | **Sometimes** | **Often** | **All the time** |
| --- | --- | --- | --- | --- | --- | --- |
| 1. | **How often does this child have difficulty completing assignments at school?** |  |  |  |  |  |
| 2. | **How often does this child have difficulty completing homework?** |  |  |  |  |  |
| 3. | **How often does this child avoid or say they do not want to do tasks that require reading or close work?** |  |  |  |  |  |
| 4. | **How often does this child fail to give attention to details or make careless mistakes in schoolwork or homework?** |  |  |  |  |  |
| 5. | **How often does this child appear inattentive or easily distracted during reading or close work?** |  |  |  |  |  |
| 6. | **How often do you worry about this child's school performance?** |  |  |  |  |  |

(Rouse *et al.*, 2009)

25/05/22

V1

References

Rouse, M., Borsting, E., Mitchell, G.L., Kulp, M.T., Scheiman, M., Amster, D., Coulter, R., Fecho, G. and Gallaway, M. (2009) ‘Academic behaviors in children with convergence insufficiency with and without parent-reported ADHD’, *Optometry and Vision Science*, 86(10), pp. 1169–1177. Available at: https://doi.org/10.1097/OPX.0b013e3181baad1

Parental Academic Behaviour Survey

Survey: 1/2/3

Date:

Participant Name:

|  |  | **Never** | **Rarely** | **Sometimes** | **Often** | **All the time** |
| --- | --- | --- | --- | --- | --- | --- |
| 1. | **How often does your child have difficulty completing assignments at school?** |  |  |  |  |  |
| 2. | **How often does your child have difficulty completing homework?** |  |  |  |  |  |
| 3. | **How often does your child avoid or say they do not want to do tasks that require reading or close work?** |  |  |  |  |  |
| 4. | **How often does your child fail to give attention to details or make careless mistakes in schoolwork or homework?** |  |  |  |  |  |
| 5. | **How often does your child appear inattentive or easily distracted during reading or close work?** |  |  |  |  |  |
| 6. | **How often do you worry about your child's school performance?** |  |  |  |  |  |

(Rouse *et al.*, 2009)

25/05/22

V1

References

Rouse, M., Borsting, E., Mitchell, G.L., Kulp, M.T., Scheiman, M., Amster, D., Coulter, R., Fecho, G. and Gallaway, M. (2009) ‘Academic behaviors in children with convergence insufficiency with and without parent-reported ADHD’, *Optometry and Vision Science*, 86(10), pp. 1169–1177. Available at: https://doi.org/10.1097/OPX.0b013e3181baad13
