# Supplementary material for: The ARUTIS Study (Anglia Ruskin University Trial of the Intuitive System): a single-centre, double-masked randomised controlled crossover trial of precision tinted lenses for visual stress: study protocol for a randomised controlled trial
Source: Trials. 2025 Dec 16;27:61. doi: 10.1186/s13063-025-09305-8 (PMC12822186; doi:10.1186/s13063-025-09305-8)
Supplement: Supplementary file 8 — Additional file 8. [file 13063_2025_9305_MOESM8_ESM.docx]

**PARTICIPANT CONSENT FORM**

**NAME OF PARTICIPANT:**

Title of the project: The ARUTIS Study (Anglia Ruskin University Trial of the Intuitive System)

Main investigator and contact details:

Lead Researcher: Zahra Nausheen Ramsahye [zr6@aru.ac.uk](mailto:zr6@aru.ac.uk)

Members of the research team:

Primary Supervisor: Peter Allen [peter.allen@aru.ac.uk](mailto:peter.allen@aru.ac.uk)

Secondary Supervisor: Nikita Thomas [nikita.thomas@aru.ac.uk](mailto:nikita.thomas@aru.ac.uk)

External Supervisor: Arnold Wilkins arnold@essex.ac.uk

Collaborator: Bruce Evans bjwe@bruce-evans.co.uk

Collaborator: Jim Gilchrist j.m.gilchrist@gmail.com

1. I agree to take part in the questionnaire. I have read the Participant Information Sheet (10.10.23 V2) for the study.

I understand what my role will be in this research, and all my questions have been answered

answered to my satisfaction.

2. I understand that I am free to withdraw from the research at any time, without giving a reason.

3. I am free to ask any questions at any time before and during the study.

4. I understand what information will be collected from me for the study.

5. For the purposes of the Data Protection Act (2018), if this project requires me to produce personal data, I have read and understood how ARU will process it.

6. I understand what will happen to the data collected from me for the research.

7. I have been told about any disadvantages or risks regarding me taking part.

8. I understand that quotes from me may be used in the dissemination of the research.

9. I have been informed how my data will be processed, how long it will be kept and when it will be destroyed.

10. I have been provided with a copy of this form and the Participant Information Sheet (10.10.23 V2).

11. I agree to my data being anonymised at the start of the clinical trial and for publishing the results of the research.

12. I understand that as the parent/guardian, I will act as a gatekeeper to this study.

Name of participant (print) ………………………………………………………………

Signed (on behalf of child if under 16 by parent/guardian) …………………………………………………….

Date……………………………….……

Email

(If you would like a report of the results of this study/ be sent a questionnaire 6 months after the trial so we can investigate long-term effects/ happy to be contacted for any further research) ………………………………………….

PARTICIPANTS MUST BE GIVEN A COPY OF THIS FORM TO KEEP

ADD DATE AND VERSION NUMBER OF CONSENT FORM.

I WISH TO WITHDRAW FROM THIS STUDY.

If you wish to withdraw from the research, please speak to the researcher or email them at [zr6@aru.ac.uk](mailto:zr6@aru.ac.uk) stating the title of the research or send them this withdrawal slip.

You do not have to give a reason for why you would like to withdraw.

When data is anonymised, it means any personal data relating to it has been permanently removed, so the researcher will not know which data belongs to you, but you are free to withdraw at any time.

Date 10.10.23

V2
