# Supplementary material for: The ARUTIS Study (Anglia Ruskin University Trial of the Intuitive System): a single-centre, double-masked randomised controlled crossover trial of precision tinted lenses for visual stress: study protocol for a randomised controlled trial
Source: Trials. 2025 Dec 16;27:61. doi: 10.1186/s13063-025-09305-8 (PMC12822186; doi:10.1186/s13063-025-09305-8)
Supplement: Supplementary file 10 — Additional file 10. [file 13063_2025_9305_MOESM10_ESM.docx]

**Section A: The Research Project**

**Title of project:**

The ARUTIS Study (Anglia Ruskin University Trial of the Intuitive System)

**Purpose of study**

Visual stress (VS) is classified as a condition that includes symptoms of visual perceptual disorders, headaches and eyestrain when viewing a repetitive pattern, including lines of text. VS is commonly mistaken as dyslexia, dyspraxia, or autism but it can happen in a general population. Students who encounter headaches, eyestrain, and visual perceptual disorders when reading are less likely to focus for prolonged periods of time.

VS testing is often carried out by optometrists but can also be carried out by other specialists in the field. Current research reveals symptoms of visual stress are alleviated by prescribing Intuitive Overlays (IO) or Precision Tinted lenses (PTL) using the Intuitive Colorimeter (IC) which was developed by Wilkins at the UK Medical Research Council (MRC).

IO are thin sheets of coloured plastic which are placed over text to reduce symptoms. PTL are spectacles with a special colour tint to reduce symptoms. The benefit of PTL is that it can be used by children more easily in a classroom setting.

We are aiming to understand how to identify and alleviate the symptoms of VS. We hope to gain a better understanding of the different types of symptoms that sufferers of visual stress experience and how these symptoms may change or improve with prescribing PTL. As part of the study, some participants may only be asked to carry out a questionnaire to ensure it is clear and easy to understand for the intended participants for the clinical trial.

**Who is the researcher?**

The lead researcher in this project is Zahra Nausheen Ramsahye BOptom (Hons) McOptom Prof cert Med Ret PGCE.

This research project is part of a PhD study.

The primary supervisor for this research project is Professor Peter Allen BSc (Hons) PhD FCOptom FHEA FAAO FEAOO.

The secondary supervisor for the research project is Dr Nikita Thomas BSc (Hons) PhD MCOptom.

**Why have I been asked to participate?**

We are recruiting subjects from the ages of 9-18 with symptoms of VS. You may also have been referred to our clinic due to reading difficulties and have not been formally diagnosed with VS.

**How many people will be asked to participate?**

The sample size for this project is around 120 participants.

**Do I have to take part?**

No. Your participation in this study is voluntary. You may withdraw at any time without giving a reason, with no consequences.

**Has the study got ethical approval?**

Yes, the study has ethical approval from an ethics committee at Anglia Ruskin University, where the trial will be taking place.

**Has the organisation where the research is being carried out given permission?**

Yes, the study will take place at Anglia Ruskin University Eye Clinic in Cambridge. The university eye clinic has given permission for the research to take place.

There is, however, no obligation placed on you by the organisation to take part, and it is your/your child’s decision whether you would like to take part in this research.

**What will happen to the results of the study?**

The results of this study will be anonymised and will only be used for scientific purposes. The results will be written up as part of a PhD thesis, published in academic journals and may be presented at academic conferences.

**Contact for further information.**

Please feel free to ask further questions by getting in touch:

Lead Researcher: Zahra Ramsahye [zr6@aru.ac.uk](mailto:zr6@aru.ac.uk)

Primary Supervisor: Peter Allen [peter.allen@aru.ac.uk](mailto:peter.allen@aru.ac.uk)

Secondary Supervisor: Nikita Thomas [nikita.thomas@aru.ac.uk](mailto:nikita.thomas@aru.ac.uk)

External Supervisor: Arnold Wilkins arnold@essex.ac.uk

Collaborator: Bruce Evans bjwe@bruce-evans.co.uk

Collaborator: Jim Gilchrist j.m.gilchrist@gmail.com

### Section B: Your Participation in the Research Project

**What will I be asked to do?**

**Visit 1:** Following obtaining consent, we will contact you to visit the Anglia Ruskin eye clinic, we will first ask you to fill out a questionnaire describing your symptoms when reading. Following this questionnaire, we will conduct an optometric eye test which can take up to 30 minutes to determine:

1. If you have a spectacle prescription
2. How well your eyes work together.
3. The health of your eyes.

As part of the eye test, we may need to use some drops to gain a more accurate measure of your/ your child's prescription. These are drops that are safe and used in a normal everyday optometric practice and can help us when prescribing spectacles. The drops may sting on insertion for up to 20 seconds and may cause slight blurring in vision for up to 6 hours. However, we will only know if this will be necessary at the time of the eye test.

There may be optometric anomalies found during the eye test that can explain the symptoms you/ your child may be experiencing. As part of the clinical trial, if the symptoms are not deemed to be caused by VS, alternative advice within the College of Optometrists Guidelines will be given. This may mean you/or your child do not continue into the clinical trial. The symptom questionnaire will be kept securely with your eye test data in the university eye clinic if you cannot continue onto the clinical trial. All eye test data is retained for a recommended 10 years in the university eye clinic as per College of Optometrists guidelines A40.

As an eye test will be performed, we will issue you a copy of your updated prescription, but this does not replace any NHS check-ups.

If, following a full eye test no optometric anomalies are found that relate to any other condition, you /your child may enter the clinical trial. Following the full eye test, we will invite you back for one more test after a 10-minute break, this is called the ‘VS test’. The VS test should take no longer than 1 hour.

The VS test will comprise of four main tests:

1. Pattern glare test: in this test, we will try and understand the symptoms you experience when reading. You will look at three images and explain the symptoms you experience when viewing each image. We will not ask you to do this test if you/your child has a history of epilepsy.
2. Rate of reading test: in this test, we will ask you/your child to read a passage of text for 1 minute. The result of this test provides us with your reading speed. The passage of text contains nonsense sentences. We will use this as the baseline for the reading speed (and will also see if IO improves your reading speed).
3. IO test: You will be presented with a combination of coloured plastic sheets to determine if these colours help the VS symptoms.

- After this part of testing, we will determine if you have symptoms on the pattern glare test, or if the coloured overlays help your symptoms. If we find a negative result with both tests, you may not be able to continue the clinical trial.
- However, we will give you the correct optometric advice for your presenting symptoms or ask you to try the coloured overlays for 3 months and book you back in at the university eye clinic if the overlays are helping your symptoms, after these 3 months you may be able to enter back onto the clinical trial.

1. Colourimetry: this is the test which determines the best colour to help your symptoms. During this test, you will be asked to look inside an illuminated box where we will present you with a different selection of colours. You will be given two colours which reduce symptoms the most.

Following this appointment, you will be given two PTLs from the colours found in the colourimetry appointment (pair A and pair B), to be worn at different times. The goal of this study is to compare which one helps your symptoms more. The results of the colourimetry test will be sent to a lab in a company called Cerium Visual Technologies. None of your personal details or data will be passed onto this company as you will be assigned a non-identifiable participant code.

This is a double-masked randomised controlled clinical trial, this means I (as the researcher and you /your child as the participant) will not know what colour will be given to you first and which one will help you more or why. We will both find out which colour is better at the final appointment, where the ‘masking’ is removed.

You/your child will then be given a symptom diary. Please complete this daily; this helps us identify how much the PTL will help your symptoms.

This is the last time you will see me until the end of the trial (visit 5), other members of the research team will deal with visits 3 and 4 to maintain the ‘masking’ aspect of the clinical trial.

**Visit 3:** You will need to then return in roughly 2 weeks for a collection appointment of pair A PTL. This appointment will only take 30 minutes. We will also perform another rate of reading test and ask to continue the symptom diary daily. We will ask you to keep these spectacles and wear them for one month when performing reading tasks.

In the last week of wear, there will be an academic behaviour survey required to be completed by the parent and the teacher. You/your child will be required to fill in the symptom questionnaire again at the end of wearing pair A PTL.

You/your child will then stop wearing and post pair A PTL back to us, we will provide you with a free postage bag.

You/your child will not be required to wear the spectacles for 1 month. However, you will be asked to continue the symptom diary.

**Visit 4:** We will contact you to book another collection appointment to pick up pair B PTL. At this visit, we will do the rate of reading test again. This appointment should take no longer than 30 minutes. We will ask you to continue the symptom dairy again every day for one month and wear pair B PTL for one month.

In the last week of wear, there will be an academic behaviour survey required to be completed by parent and teacher. You/your child will be required to fill in the symptom questionnaire again at the end of wearing pair B PTL.

**Visit 5:** After 1 month of wearing pair B, we will contact you for an appointment. This visit is where we will compare which PTL is better for you/your child and reduces VS symptoms the most. This is where the clinical trial ends and the research team will start to analyse all the data. You/your child are free to keep the PTL which reduces VS symptoms the most. This last appointment should last around 1 hour.

**PPlease see the below flow chart for a visualisation of the study.**

**
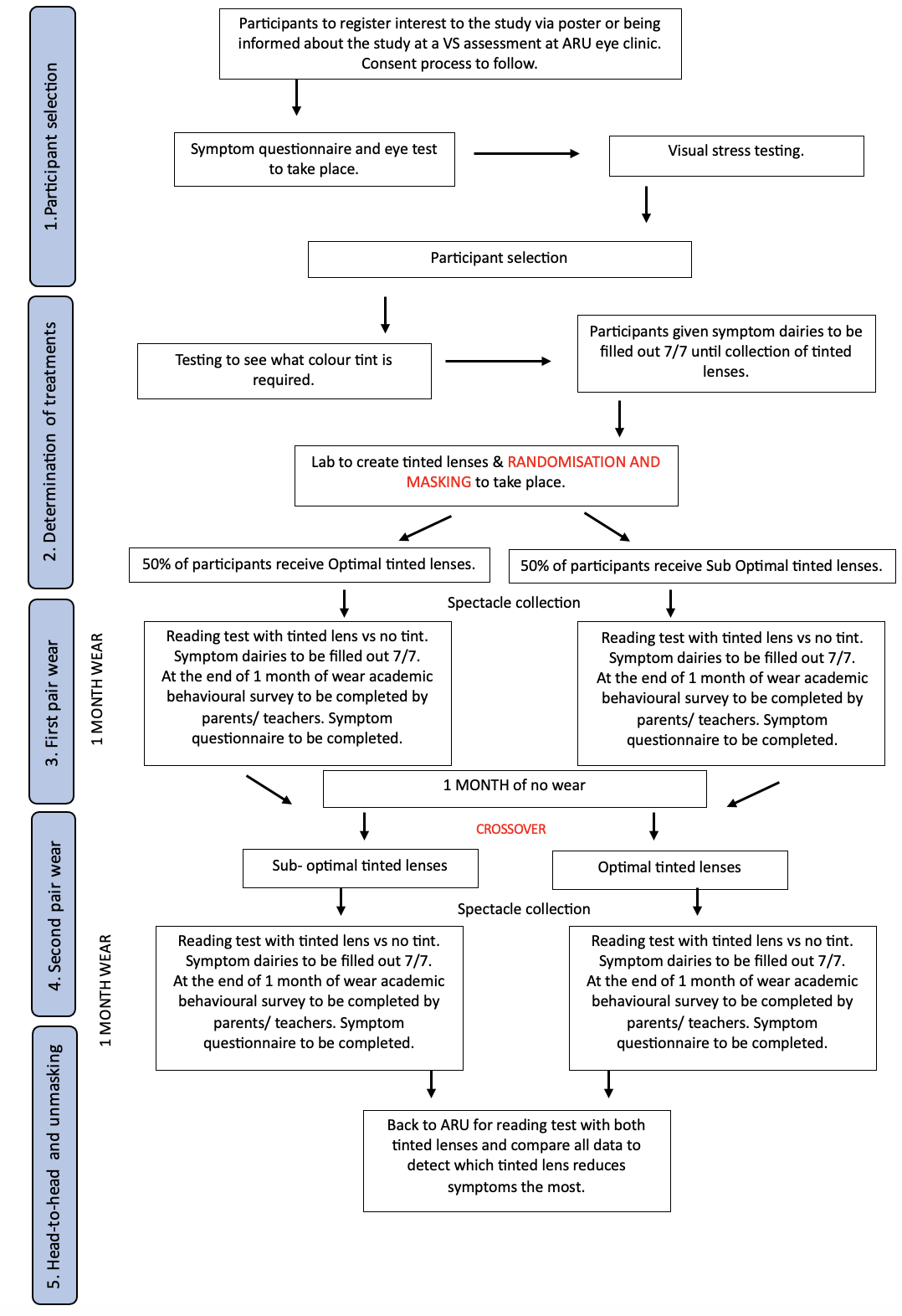
**

**In relation to this specific research project, we need to make you aware of the following:**

| ☐ | We do not need your personal data at any stage of this research project | | |
| --- | --- | --- | --- |
| We are responsible for the personal data you give to us as a: | | | |
| ☐ | **Data Controller**  (We are in sole control over the research) | Who are we? | Zahra Ramsahye  Peter Allen  Nikita Thomas |
| ☐ | **Joint Controller**  (Where ARU and another organisation are working together on research) | with: |  |
| ☐ | **Data Processor** (Where the data will belong to another organisation and ARU is being engaged under contract/agreement to conduct the research and provide an outcome but has no rights over the personal data) | on behalf of: |  |

**I will be asking you for the following information only for the eye test purpose:**

| **Personal Data** | | | | **Sensitive Personal data** | |
| --- | --- | --- | --- | --- | --- |
| ☐ | Name/Contact details | ☐ | Image | ☐ | Racial/Ethnicity data |
| ☐ | Age | ☐ | Experiences | ☐ | Political/Religious beliefs |
| ☐ | Address/location data | ☐ | Opinions | ☐ | Trade Union membership |
| ☐ | Employment & Earnings | ☐ | [Other] | ☐ | Genetic/Biometric data |
| ☐ | ID Numbers (e.g. NHS) | ☐ | [Other] | ☐ | Health |
| ☐ | Online identifier | ☐ | [Other] | ☐ | Sex life/orientation data |

We do not intend to collect data held about participants from existing records. All data collected and stored will comply with the UK GDPR, Data Protection Act (2018).

**What will happen to your data?**

Your confidentiality will be maintained during this clinical trial. The purpose of collecting eye test data is to allow us to investigate any other causes of the symptoms you may be experiencing. The consent form is the only other record in this study containing your personal details, the consent form will be kept securely in a locked cupboard which is used to store research materials at the School of Optometry and Vision Sciences Anglia Ruskin University. We will use a non-identifiable participant code to label the data required for the clinical trial and will be used as part of a PhD thesis.

We will anonymise your data for the clinical trial and for any research purpose. Anonymisation refers to the process of removing personal identifiers that may lead to a person being identified from that information or combined with other information.

A participant code will be assigned to you after the eye test has taken place. This means you can enter the clinical trial. You will need this code when you come to collect your PTLs. We will not link the code back to your personal data at the end of the study. All data collected after this point is required for the clinical trial and will be fully anonymised from this point forward with the participant code.

The data processing and analysis will be carried out in the UK and will comply with UK law. All data will be securely held at the university eye clinic. Any data which is shared with the primary, secondary, or external supervisors or research collaborators will be anonymised.

All data will be anonymised within the PhD thesis. However, even though we will make every attempt to ensure your data is anonymous, there is a chance you could be identified, by peers or colleagues for example.

We may use quotes from your time during this clinical trial in the PhD thesis which may increase the likelihood that you could be identified. However please read the participant consent form and sign if you are happy for us to do this.

If you are happy to provide an email we will send you a questionnaire 6 months after the clinical trial has ended to discuss the long-term effects with the PTL / contact you for any further research (you may decline this).

**Will I be reimbursed for travel expenses?**

No, we will not be able to reimbue travel expenses.

**Will I receive any payment to take part in the research?**

You will not receive payment; however, you will be able to keep the PTL which helps your/ your child's symptoms the most. The usual cost of the PTL from the Anglia Ruskin University eye clinic is £150. You are free to pick your own frame from the selection we have and there will be no cost for this. However, you are also free to choose your own frame if you do not like the selection we have, although this will be at your own cost.

**Are there any possible disadvantages or risks to taking part?**

As this is a non-invasive clinical trial there are minimal disadvantages and risks to taking part.

The main disadvantage is the PTL not improving symptoms of reading speed or poor/uncomfortable fitting frames. If at any point during the clinical trial, you or your child’s frames do not fit correctly, there will be no charge for adjustment from the university eye clinic.

There are no other risks such as physical or mental harm. All testing complies with the College of Optometrist’s guidelines and the General Optical Council.

You can withdraw from the clinical trial at any point you wish for whatever reason even if not stated on this information sheet.

Agreement to participate in the study does not affect you or your child’s legal rights.

**What are the likely benefits of taking part?**

The benefits of taking part can range from a reduction in headaches, eye strain, visual perceptual distortion, and improved reading speed. However, as the symptoms of VS can range in every participant, therefore the improvement will also be very induvial. There will also be an improved understanding of the symptoms of VS and how to best diagnose VS.

**Can I withdraw at any time, and how do I do this?**

You or your child can withdraw from the study at any time and without giving a reason. We understand participants may not feel comfortable telling us directly in person that you would no longer like to take part in your research. Therefore, you may email the lead researcher, any supervisor or collaborator.

Questionnaires and symptom dairies are an important part of this clinical trial as we need to analyse the data. You need to let us know at the start of the clinical trial at visit 1 if you do not wish to answer questionnaires or fill out the symptom diary, unfortunately, we will not be able to put you onto the clinical trial if this is the case.

**What will happen to my data?**

| **Our general privacy notice explaining our use of your personal data for research purposes is available here:**  <https://www.aru.ac.uk/privacy-and-cookies/research-participants>  **Please visit this link for information about how long we keep your data, how we keep your data secure, how you can exercise your rights over your data, and make a complaint about our use of your data.** |
| --- |

**Can I withdraw my data from the study?**

The information I collect from you/your child as part of the clinical trial will be anonymous. This means that I won’t be able to remove your data because I won’t know which data belongs to you/your child.

**Whether there are any special precautions you must take before, during or after taking part in the study?**

No.

**Will I pass on to anyone else what you have told me?**

No, all records of eye test examinations and data will remain in the university eye clinic. All data is anonymised and will only be analysed for this clinical trial, the data will remain on the university eye clinic computer.

However, if at any part of the clinical trial, you or your child disclose information which I feel puts anyone at risk, reveals anything of an illegal nature or reveals anything of an unprofessional nature, I will need to disclose this information to the ethics committee and act in your/your child’s best interest as a registered health care professional.

**Summary of research findings**

The results of this research will form part of a PhD thesis and we also aim to publish them in scientific journals. If you would like to hear about the results of the study, please provide an email or postal address on the back of the consent form.

**Contact details for complaints**

If you have any complaints about the study, please speak to the lead researcher or any member of the supervisory team in the first instance, to try and reach an informal resolution. Below is also access to details about ARU’s complaints procedure.

Email address: [complaints@aru.ac.uk](mailto:complaints@aru.ac.uk)

Postal address: Office of the Secretary and Clerk, ARU, Bishop Hall Lane, Chelmsford, Essex, CM1 1SQ.

**Version control**

Date 10^th^ October 2023

Version 2

PARTICIPANTS SHOULD BE GIVEN A COPY OF THIS TO KEEP,

TOGETHER WITH A COPY OF THE CONSENT FORM.
